# Porphobilinogen Synthase from the Butterfly, *Pieris brassicae*: Purification and Comparative Characterization

**DOI:** 10.1673/031.007.6201

**Published:** 2007-12-17

**Authors:** Roland Rilk-van Gessel, Hartmut Kayser

**Affiliations:** Department of General Zoology and Endocrinology (Biology I), University of Ulm, Albert-Einstein-Allee 11, D-89069 Ulm, Germany

**Keywords:** Tetrapyrrole biosynthesis, enzyme purification, inhibitors, preparative native electrophoresis, immunoprecipitation, insect phylogenesis

## Abstract

Porphobilinogen represents a key building block of tetrapyrroles serving as functional ligands of many vitally important proteins. Here we report the first purification of porphobilinogen synthase (PBGS) from whole insects by sequentially employing two modes of native electrophoresis on polyacrylamide gels subsequent to more conventional procedures. Using adults of *Pieris brassicae* L. (Lepidoptera: Pieridae) we achieved ∼10,000-fold purification with final yields of up to 25% of electrophoretically pure PBGS with a specific activity of ∼160 µmol PBG h^-1^ mg^-1^ at 37°C and an affinity of 0.36 mM to its substrate 5-aminolevulinic acid. Enzyme activity was inhibited by the substrate mimics, levulinic acid and succinylacetone, and by chelating agents. PBGS behaved as a relatively heat-stable octameric complex of 292.3 kDa composed of 36.5 kDa subunits. Most general features of this insect PBGS were comparable to those published for other animal PBGS enzymes, while remarkable differences were found to the reported recombinant *Drosophila* enzyme. Moreover, rabbit antiserum directed against purified *Pieris* PBGS revealed significant immunological differences among insect PBGS enzymes from a wide range of orders contrasting to the overall evolutionary conserved features of this enzyme.

## Introduction

Tetrapyrroles are synthesized in all biological kingdoms to provide the molecular basis for respiration, photosynthesis and the activity of many enzymes (Warren and Scott 1990). Porphobilinogen (PBG) represents the basic tetrapyrrole building block that is produced in the cytosol by the asymmetric condensation of two molecules of 5-aminolevulinic acid (ALA) in the common first step of tetrapyrrole biosynthesis catalysed by porphobilinogen synthase (PBGS, EC 4.2.1.24, or 5-aminolevulinate dehydratase). Hence, PBGS enzymes represent a ubiquitously present and evolutionary conserved family of proteins while differing in some features such as metal requirement or pH optima (Jaffe 2000, 2004).

While PBGS enzymes from mammalian, plant, yeast and bacterial origin have been well studied those from insects and other invertebrates are poorly known to date. The only known invertebrate PBGS sequence is that from *Drosophila* in which the bacterially expressed enzyme has been studied recently (Kundrat et al. 2003). This is surprising as many insects, lepidopteran species in particular, do produce fairly large quantities of open-chain tetrapyrroles, the heme-derived bilins that serve as specific ligands of biliproteins some of which have been identified as members of the lipocalin protein family (Kayser 2005; Ganfornina et al. 2006). As an approach to understanding the synthesis and the function of these biliproteins, the developmental variation of PBGS activity and of the levels of ALA and PBG, its substrate and product, is being studied in *Pieris brassicae* L. (Lepidoptera: Pieridae), a lepidopteran insect that synthesizes under a developmental regime a bilin-binding protein in significant quantities (Kayser et al. 2005). One goal of this project is to purify PBGS from this insect to characterize an invertebrate representative of this enzyme family.

Here, we report the first purification of native PBGS from whole insects in electrophoretically homogeneous quality and in high yield. While the early steps of purification could be well performed using procedures frequently employed to purify PBGS enzymes, other methods had to be examined to obtain the insect enzyme in pure form and in reasonable quantity. Two different modes of native electrophoresis in polyacrylamide gels were sucessful. This strategy may also be applicable to PBGS from other sources that are low in PBGS activity. Antibodies were produced against purified *P. brassicae* PBGS to examine how conserved at an immunological level this butterfly enzyme behaved versus its counterparts from other insects and vertebrates. Such a study has not yet been performed with PBGS from invertebrates.

## Materials and Methods

### Chemicals

5-Aminolevulinic acid (ALA) hydrochloride and D(+)-lactose monohydrate were from Fluka-Aldrich (www.sigmaaldrich.com). Levulinic acid was obtained from Merck (www.merck.com). Succinylacetone (4,6-dioxo-heptanoic acid) was purchased from United States Biochemicals (www.usbweb.com). Low-molecular weight markers for polyacrylamide gel electrophoresis (PAGE) were from Pharmacia (www.Pharmacia.com). Reference proteins for gel filtration, chemicals for electrophoresis and polyethylene glycol (PEG) 6000 were from Serva (www.serva.de). Bovine serum albumin was obtained from Sigma. TEAE-cellulose was from Schleicher and Schuell (www.s-and-s.com), and HA-Ultrogel was from LKB-Pharmacia. Standard chemicals were purchased from either Fluka-Aldrich or Merck.

### Insects

The stock of *P*. *brassicae* was maintained on a semisynthetic diet under non-diapausing conditions at 21°C, as in former work (Kayser 1984). For enzyme purification, adult insects were collected between 6 and 12 h after emergence and stored at -20°C. The other insects and the vertebrate tissues were obtained from in-house stocks and commercial breeders, respectively.

### Determination of PBGS activity

PBGS activity was assayed as in previous work (Kayser et al. 2005) with minor modifications. Briefly, enzyme activity was determined in a total volume of 1 ml of assay medium containing of 0.1 M sodium phosphate (pH 6.8), 2 mM DTT and 3.5 mM ALA. Incubations were carried out at 50°C (for enhanced sensitivity) for 1 h, if not stated otherwise. The PBG product was quantified with modified Ehrlich's reagent measured at 555 nm. The enzyme concentration was always kept high enough to prevent loss of activity due to subunit dissociation; the lower limit for A555 nm under standard conditions was 0.2, as found in initial studies (not shown). PBGS activity was expressed as µmol; produced in 1 h at 50°C. Specific activity was calculated per mg of protein. PBGS activity at 37°C was calculated from the 50°C-data by division by 1.75; this factor was obtained from a study of the temperature effect on the activity of PBGS from *P*. *brassicae* (see Figure 2A).

The enzymatic properties of PBGS were studied using a partially purified (∼600-fold) preparation under standard conditions as described. To determine substrate affinity incubations were terminated after 15 min. Inhibitors were dissolved in assay medium; if necessary, the pH of the stock solutions was brought to 6.8 with NH4CO3 solution. Inhibitor assays were performed for 60 min without pre-incubation.

### Preparation of PBGS extract

Frozen butterflies were homogenized in batches of about 100 g (about 600 insects) in cold medium A, consisting of 20 mM sodium phosphate (pH 6.8) and 20 mM 2-mercaptoethanol, at a ratio of 5.5 ml/g insect weight. A small quantity of N-phenylthiourea was added to prevent phenoloxidase activity. The homogenates were centrifuged at 27,000xg for 20 min at 2°C (Sorvall RC-2B; rotor GSA) and the resulting supernatants were filtrated through silanized glass wool. Aliquots of 200 ml of the turbid extracts were heated to 58°C under steady stirring in a water bath maintained at 65°C. When 58°C was reached, the extracts were immediately cooled down to below 10°C in an ice bath. After centrifugation, clear supernatants were obtained for enzyme purification. Calculations of enzyme yields and purification factors were based on these heat-treated extracts (see Table 1).

### Fractional precipitation

First, the heat-treated extracts were fractionated by ammonium sulfate added in solid form to successively reach 42% and 50% saturation at 0°C each step being followed by centrifugation at 40,000 × g for 20 min (Sorvall RC-5B with rotor SS34). The 50%-sediment containing PBGS was dissolved in a small volume of medium B consisting of 20 mM sodium phosphate (pH 6.8) and 1 mM DTT and next subjected to further fractionation using PEG 6000. PEG was first applied at a concentration of 7% (w/v) at 0°C. Following centrifugation, PEG concentration was increased by the addition of 27 g/100 ml of supernatant at room temperature (to increase solubility). After 1 h, the precipitate was collected at 40,000xg for 15 min and dissolved in medium B.

### Column chromatography

The dissolved PEG-sediment was applied to a column of TEAE-cellulose (1.6 g/100 g of insects) equilibrated with medium B. One bed volume of medium B supplemented with 5 mM 2-mercaptoethanol was applied to the column prior to sample loading. This measure was essential for a high recovery of active PBGS; most likely, the effect was due to protection of active site thiol groups. The column was then washed with medium B containing 50 mM NaCl until absorbance at 280 nm of the effluent was constantly low. PBGS was eluted from the column employing a gradient of 50 to 450 mM NaCl in medium B at a rate of 0.25 ml/min. Enzyme activity was obtained in a total volume of about 30 ml (about 10 fractions) and directly pumped onto a column of Hydroxyapatite-Ultrogel (HA-Ultrogel; 1.5 × 4 cm, *www.pall.com*) also equilibrated with medium B. After thorough washing the column with the same medium, PBGS was eluted with 60 mM phosphate buffer (pH 7.7); this step mode proved to be superior to gradient elution (20 to 400 mM sodium phosphate; pH 6.8; 1 mM DTT). PBGS was almost quantitatively obtained as a single peak in a volume of 10 to 12 ml that was reduced to about 1 ml by ultrafiltration followed by dialysis against the sample buffer for electrophoresis using an Amicon 8MC filtration unit with a PM-30 membrane.

### Preparative native polyacrylamide gel electrophoresis (PAGE)

Preparative PAGE under native conditions was performed first in 3-mm thick slab gels run in a Protean Slab Cell apparatus (BioRad, *www.bio-rad.com*). The gel system consisted of a 3% concentrating gel (pH 6.9), a 5.25% separation gel (pH 8.9) and a 25% lower (blocking) gel (pH 8.1). A channel between the separation and the lower gel allowed the continuous elution of proteins leaving the separation gel. This channel was prepared by pipetting a 40% (w/v) sucrose solution onto the polymerized lower gel before carefully filling the acrylamide solution for the separation gel onto the sucrose solution. The sample volumes varied between 2 and 3 ml containing a maximum of 25 mg protein from the preceding hydroxyapatite step. Tris/glycine of pH 8.8 was used as cathode buffer and Tris/chloride of pH 8.3 as anode buffer. The gels were run at 20 mA during stacking and 40 mA during separation. Electrophoresis was terminated after a total running time of about 7 h. The eluted proteins were collected in 2-ml fractions by continuous pumping of elution buffer (0.2 M Tris/chloride; pH 8.3; 1 mM DTT) through the channel at a rate of 0.5 ml/min. Fractions were assayed for PBGS activity and for protein (280-nm absorbance). Active fractions were pooled, concentrated down to a volume of 1 to 2 ml by ultrafiltration, and then dialyzed against 10 volumes of sample buffer.

The second preparative native PAGE was performed in 7.5% rod gels of 1-cm diameter using the same electrode buffers as for the slab gels. Running conditions were 4 mA during concentration and 9 mA during separation. The gels were then cut into 1-mm slices and PBGS was recovered in a small volume (approx. 0.3 ml) by electrophoresis performed at 1 mA at 4°C overnight using a lab-made device. If dialyzed against medium supplemented with 1% lactose, PBGS could be stored frozen over months without loss of activity.

### Analytical polyacrylamide gel electrophoresis (PAGE)

To guide PBGS purification, samples from every step were subjected to native analytical PAGE in 7.5% gels, as described for system 1a by Maurer (1971). Proteins were stained with Coomassie Blue G-250 and contrasted in 5% acetic acid. To detect PBGS activity, the gels were incubated with 2.7 mM ALA in assay buffer for 15 min at 50°C followed by staining of produced PBG with modified Ehrlich's reagent, as described above.

The molecular mass of the holoenzyme was determined from the slope of its mobility on native PAGE according to Hedrick and Smith (1968) using the buffer system from Davis (1964). The gel concentrations were 4.5, 6.0, 7.5 and 9.75%. Reference proteins were urease (483 kDa), pyruvate kinase (238 kDa), aldolase (147 kDa), lactate dehydrogenase (140 kDa), citrate synthase (90.5 kDa) and bovine serum albumin (67 kDa). Proteins and PBGS activity were stained as described above.

Protein subunits were studied by SDS/urea-PAGE (Laemmli 1970; Merle and Kadenbach 1980). The molecular-weight marker proteins were phosphorylase B (94 kDa), bovine serum albumin (67 kDa), ovalbumin (43 kDa), carboanhydrase (30 kDa), trypsine inhibitor (20.1 kDa) and lactalbumin (14.4 kDa). Proteins were stained with Coomassie Blue R-250.

### Protein determination

To remove interfering substances and to concentrate dilute samples, proteins were first precipitated at 6% (w/v) trichloroacetic acid in the presence of desoxycholate (Bensadoun and Weinstein 1976). The resulting pellets were dissolved in 50 µl of 0.2 M NaOH containing 2% (w/v) SDS for quantitative determination of protein with the Folin-reagent (Hartree 1972, modified). Bovine serum albumin was used as a standard under the same conditions. At the two final steps of purification, protein was determined from the absorbance at 280 nm. An extinction coefficient of 0.864 [0.1%^-1^ 1cm^-1^] was obtained from an aqueous solution of the purified PBGS after dialysis against water and lyophilisation.

### Immunological studies

Antibodies against purified PBGS (total of ∼100 µg) from *P*. *brassicae* were produced in rabbits according to standard procedures. Control sera were obtained from non-immunized rabbits. Heat-treated PBGS extracts from various insects and from vertebrate livers (0.25 to 0.4 g/ml medium A) were obtained as described above for *P. brassicae.* Based on initial tests to optimise conditions, the antisera and control sera, respectively, were diluted with assay buffer 1:8 (v/v), mixed with an equal volume of the respective heated PBGS extract and incubated for 42 h at 5°C. After removal of the immunoprecipitate by centrifugation, 250 µl of supernatant was incubated with 750 µl of assay medium for 2 h to determine residual PBGS activity. From this value, the immunoprecipitated PBGS activity as percentage of the activity of controls without antiserum was calculated as a measure of immunological relatedness.

## Results

### Purification of PBGS

Data of a representative purification of PBGS from *P*. *brassicae* are summarized in Table 1.

The turbid raw extracts contained PBGS activity in the range of 0.6 µmol h^-1^ and total protein of about 40 mg/g insect mass. Because PBGS is known as a fairly heat stable enzyme (e.g., Coleman 1970), confirmed also in the present study, the turbid extracts were cleared by heating to 58°C.

PBGS was precipitated from the heat-treated extracts with ammonium sulfate at concentrations between 42 and 50% saturation at 0°C providing typical yields of about 90%. The pellet was dissolved in medium B to replace 2-mercaptoethanol for DTT. This crude PBGS preparation was subjected to fractional precipitation with PEG 6000 performed first at 10% PEG at 0°C followed by 30% at room temperature. PBGS was recovered in the pellet at 90 to 95% yield and again taken up in medium B.

**Figure 1.**
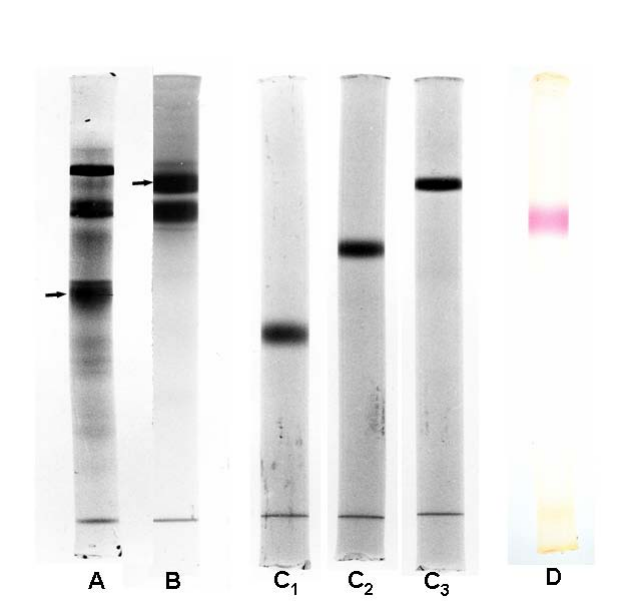
Analysis by native PAGE of the purification of PBGS from *P*. *brassicae.* A - After chromatography on HA-Ultrogel; 5.25% gel. B - After preparative PAGE 1 (slab gel); 7.5% gel. C - after preparative PAGE 2 (rod gel): gel concentrations were 5% (C1), 6.25% (C2) and 7.5% (C3), respectively. In C, each gel was loaded with 10 µg of purified PBGS. Gels A-C were stained with Coomassie Brilliant Blue G-250. The arrows in A and B mark the PBGS band identified by in situ incubation of the gels with substrate followed by product staining with Ehrlich's reagent, as shown in D (7.5% gel).

**Table 1.**
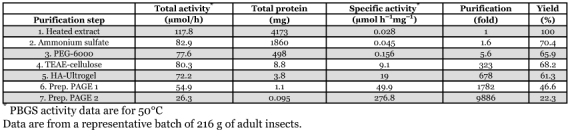
Purification of PBGS from *P*. *brassicae.*

**Figure 2.**
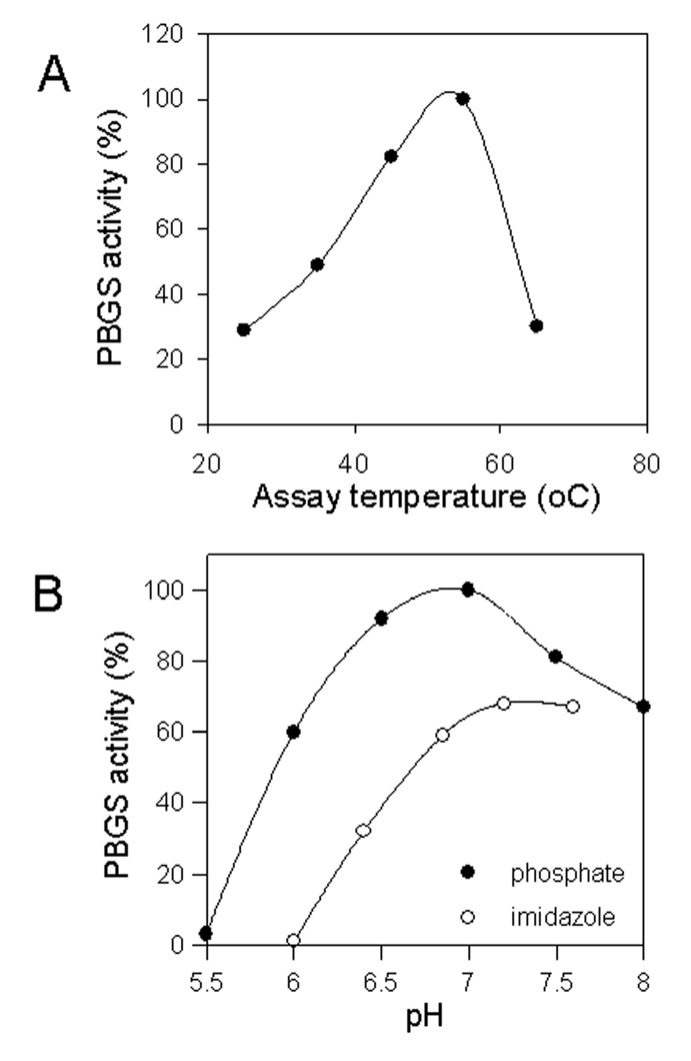
Dependence of PBGS activity on (A) temperature (in 100 mM sodium phosphate, 10 mM 2-mercaptoethanol; pH 6.8) and (B) on pH of phosphate buffer (100 mM sodium phosphate, 10 mM 2-mercaptoethanol) and imidazole buffer (100 mM imidazole/HCl, 2 mM DTT), respectively, both assayed at 50°C.

The subsequent chromatography on TEAE-cellulose provided an about 60-fold increase in specific activity of PBGS and almost quantitative recovery. The enzyme was gradient-eluted as a relatively sharp single peak at a NaCl concentration around 200 mM in a volume of about 30 ml. The active fractions from the TEAE-column were further purified on a column of HA-Ultrogel equilibrated with medium B. PBGS was quantitatively adsorbed to the hydroxyapatite. After washing with medium B, PBGS was step-eluted by 60 mM sodium phosphate buffer (pH 7.7) containing 1 mM DTT. Recovery of PBGS activity from the hydroxyapatite matrix was about 90% with an about 2-fold increase of specific activity. Analysis of this preparation by native PAGE revealed the presence of a number of strong protein bands one of which could be clearly identified as PBGS by *in* *situ* staining of activity (not shown)(Figure 1A).

**Figure 3.**
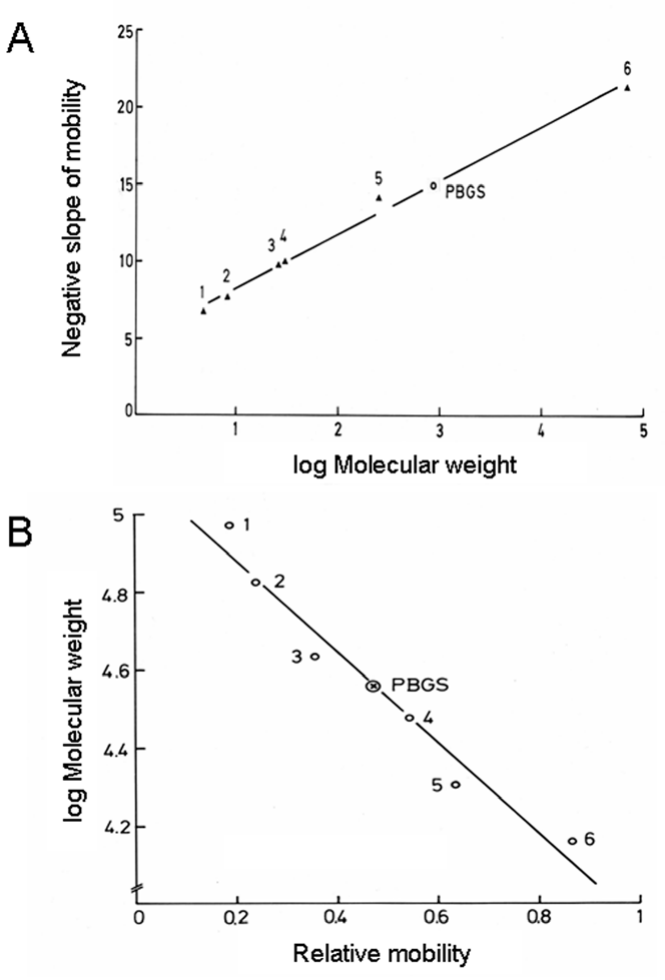
Determination of the molecular mass of PBGS from *P*. *brassicae.* A - Native PAGE. The reference proteins were bovine serum albumin (1), citrate synthase (2), lactate dehydrogenase (3), aldolase (4), pyruvate kinase (5) and urease (6). A molecular weight of 292'300 was obtained for the native complex of PBGS. B SDS/urea-PAGE. The reference proteins were phosphorylase B (1), bovine serum albumin (2), ovalbumin (3), carboanhydrase (4), trypsine inhibitor (5) and lactalbumin (6). A molecular weight of 36'000 was obtained for the subunits of PBGS.

Next, preparative native PAGE was employed in a 5.25% slab gel (PAGE 1 in Table 1). PBGS was eluted as a broad protein peak usually containing >70% of the applied enzyme activity with a 2- to 3-fold higher purity. Analytical native PAGE using a high protein load revealed the presence of two prominent protein bands or zones that appeared to contain more than one protein (Figure 1B): the upper band contained PBGS as revealed by activity staining (not shown); the lower band was already visible during electrophoresis as a brownish band that lacked PBGS activity. Consequently, a second preparative electrophoresis was performed using a native 7.5 % rod gel (PAGE 2 in Table 1). The gel slice containing active PBGS was electro-eluted. Though about half of the enzyme activity was lost, this final purification step provided electrophoretically pure PBGS with a specific activity of ∼280 µmol h^-1^ mg^-1^ at 50°C, corresponding to a calculated value of ∼160 µmol h^-1^ mg^-1^ at 37°C. The purity of the enzyme was demonstrated by electrophoresis in native gels of different concentrations (5, 6.25, and 7.5%: Figure 1C1–3) and in SDS gels (see below). All experiments revealed a single protein band that represented active PBGS enzyme, as shown by in situ staining (Figure 1D). In the various PBGS preparations, overall yields of 20 to 25% and purification factors around 10,000 were obtained.

**Figure 4.**
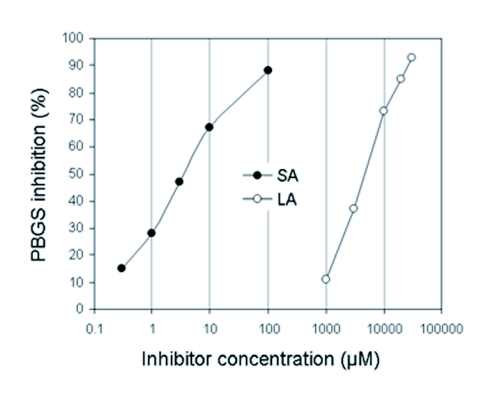
Inhibition of PBGS from *P. brassicae* by levulinic acid (LA) and succinylacetone (SA). The experiments were performed under standard conditions for 1 h without pre-incubation of the inhibitors.

## Characterization of PBGS

### Effect of temperature

In 100 mM sodium phosphate (pH 6.8) supplemented with 10 mM 2-mercaptoethanol, the temperature optimum for the *Pieris* PBGS from both adults insects and last instar larvae was at ∼55°C in 1-h assays (Figure 2A). On this basis, PBGS activity was routinely determined at 50°C for higher sensitivity compared to the commonly used 37°C. A short incubation at 58°C did not result in any significant loss of activity and was therefore employed as an initial step of purification.

### Effect of pH, buffer system and chelating agents

At the standard assay temperature of 50°C, the optimum of PBGS activity was at pH 6.8 in 100 mM sodium phosphate containing 10 mM 2-mercaptoethanol, and at pH 7.1 in 100 mM imidazole/HCl in the presence of 2 mM DTT (Figure 2B). Though the pH optima in the two buffers differed only slightly, PBGS activity was lower by 32% in imidazole buffer compared to phosphate buffer, at the respective pH optima. When exposed to pH < 6 in phosphate buffer, PBGS activity was irreversibly lost. EDTA inhibited PBGS by 67% at 0.1 mM and 78% at 1 mM (data not shown). Another chelating agent, 8-hydroxy quinoline-5-sulfonic acid, was only weakly inhibitory (23% at 1 mM; data not shown).

### Molecular mass

Native PAGE performed at different polyacrylamide concentrations revealed a molecular mass of 292.3 kDa for the purified PBGS (Figure 3A) as well as for a partially purified preparation analyzed by in *situ* activity staining. All samples displayed a single band of active PBGS. Under denaturing conditions (SDS/urea), a single protein band was also observed corresponding to 36.5 kDa (Figure 3B). Hence, PBGS from *P. brassicae* apparently represents an octameric complex of equally sized subunits, most likely a homo-octamer.

**Figure 5.**
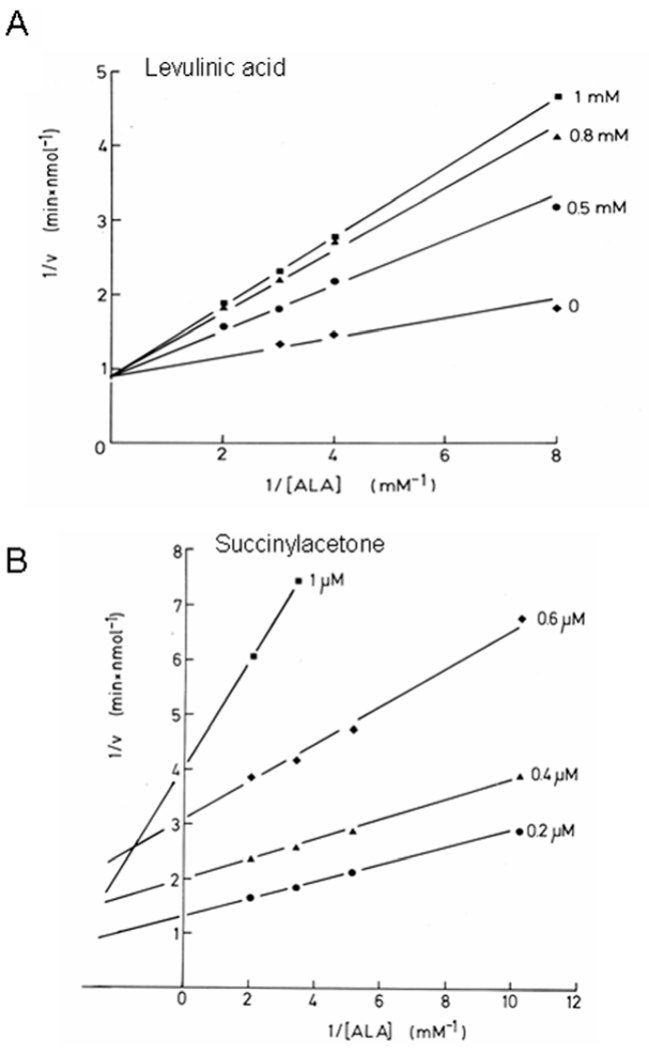
Lineweaver-Burk analysis of the inhibition of PBGS from *P. brassicae* by (A) levulinic acid and (B) succinylacetone.

### Affinity to substrate and inhibitors

The Lineweaver-Burk plot (not shown) for the PBGS substrate ALA was linear and provided a *Km* value of 0.36 mM. The substrate mimics levulinic acid and succinylacetone inhibited the enzyme with IC50 values of 3.4 mM and 4.5 µM, respectively (Figure 4). Dixon plot analysis of kinetic data for levulinic acid revealed a *K*_i_ value of 0.34 mM (not shown). The Lineweaver-Burk plot revealed a competitive mode of inhibition for levulinic acid (Figure 5A). By contrast, the plot for inhibition by succinylacetone suggested an uncompetitive mode at low (<0.5 µM) concentrations that apparently changed to a mixed non-competitive mode at higher concentrations (Figure 5B). A more detailed analysis of this observation was not attempted during this study.

**Table 2.**
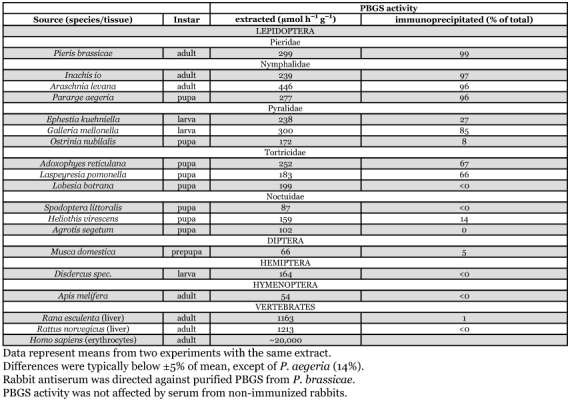
Immunoprecipitation of PBGS activity from insect and vertebrate sources.

### Immunological studies

As summarized in Table 2, antiserum directed against pure *Pieris* PBGS also precipitated PBGS from all examined members of the nymphalid lepidopteran families (*Inachis io*, *Araschnia levana*, *Pararge aegeria*) to a degree comparable to the enzyme from *P*. brassicae; only 4% or less of PBGS activity remained in the supernatant. Unexpectedly, the extracts from the examined pyralid and tortricid moths (three species of each) responded rather heterogeneously to the antiserum: while PBGS from *Galleria mellonella*, *Adoxophyes reticulana* and *Laspeyresia pomonella* was only partially recognized (66 to 85%), the enzymes from *Ephestia kuehniella*, *Ostrinia nubilalis* and *Lobesia botrana* showed very little, if any, cross-reaction, i.e. almost all PBGS activity (73 to 116%) remained in solution. Similarly, PBGS activity from all three noctuid moths examined (*Spodoptera littoralis*, *Heliothis virescens* and *Agrotis segetum*) was not or only marginally precipitated by the antiserum. Negative results were also obtained with PBGS from all other non-lepidopteran species representing dipteran (*Musca domestica*), hemipteran (*Disdercus* spec.) and hymenopteran (*Apis melifera*) insects, as well as with PBGS from vertebrate livers (frog and rat) and from human erythrocytes.

## Discussion

### Purification of *Pieris* PBGS

The PBGS from *P*. *brassicae* is the first PBGS isolated from whole insects as a native enzyme. Because published purification strategies (e.g., Coleman 1970; Anderson and Desnick 1979; Gibbs et al. 1985) were found not to be well suited to obtain a pure preparation, a new protocol was developed that may also work with PBGS from other insects or other sources of low PBGS activity or limited amounts of material. The purification scheme is characterized by high recoveries, typically in the 90% range, at all steps except for the final electrophoresis (PAGE 2), providing high final yield (up to 25%) of electrophoretically pure and active PBGS.

The *Pieris* enzyme exhibited some chromatographic behaviour that was unexpected in the light of the reportedly conserved nature of PBGS. While recombinant PBGS from *Drosophila,* the only other insect PBGS studied, could be readily purified on phenyl sepharose and on octyl sepharose (Kundrat et al. 2003), the *Pieris* enzyme was completely retained on the phenyl matrix under comparable conditions that were used to elute the fly enzyme (data not shown). On the other hand, the more lipophilic octyl sepharose could be successfully applied to *Pieris* PBGS. However, this matrix was not included in the final purification protocol because of its low efficiency (data not shown). With respect to octyl sepharose, PBGS from *Pieris* behaved similarly to the human enzyme (Anderson and Desnick 1979). In conclusion, octyl sepharose may be generally applicable in purification protocols of PBGS enzymes, while chromatographic differences may be encountered with phenyl sepharose. It may be speculated that this differential behaviour of PBGS enzymes is due to differences in the amino acids present at the protein surface that come into contact with the chromatographic matrix and which therefore determine the binding behaviour. In particular, the N-terminal arms of about 40 residues, which represent the most varied sequence stretch and wrap around neighbouring subunits at the surface of the octameric complex (cf. Erskine et al. 2001) may be relevant in this respect.

### Characterization of *Pieris* PBGS

Based on the molecular mass of *Pieris* PBGS of 292.3 kDa as determined by electrophoresis under native conditions and the value of 36.5 kDa obtained in the presence of SDS/urea, the *Pieris* enzyme represents an octameric complex composed of subunits of apparently equal size. This is in agreement with the general view of PBGS enzymes as homo-octamers (Jaffe 2000, 2004). Exceptions are the recently described rare human PBGS allele forming a hexamer with altered catalytic properties (Breinig et al. 2004) and the also hexameric enzyme from the photosynthetic bacterium *Rhodobacter capsulatus* (Bollivar et al. 2004). Furthermore, stability to heat and a pH optimum around neutrality of the *Pieris* PBGS is also in the range found with other metazoan PBGS forms differing from the basic pH optima of the enzymes from plants, fungi and bacteria. Remarkably, however, the recombinant form of PBGS from *Drosophila* differed from the present butterfly enzyme in exhibiting a pH optimum around 8 (Kundrat et al. 2003). All animal PBGS enzymes are inhibited by chelating agents in agreement with having catalytically active zinc bound by essential cysteines (Jaffe 2004) that make PBGS enzymes sensitive to oxidation.

The *K*_m_ value of 0.34 mM for ALA of the *Pieris* enzyme is also in the range of values published for other PBGS enzymes (e.g., Cheh and Neilands 1976; Coleman 1970), including the *Drosophila* enzyme for which a *K*m of ∼0.1 mM has been reported (Kundrat et al. 2003). The Lineweaver-Burk plot for PBGS from *Pieris* was linear, demonstrating normal Michaelis-Menten kinetics under our experimental conditions (cf., Stauffer et al. 2001). The specific activity of ∼280 µmol h^-1^ mg^-1^ at 50°C corresponds to ∼160 µmol h^-1^ mg^-1^ at 37°C at which PBGS enzymes are commonly studied. This value is unusually high for an animal PBGS. Typical values for the enzyme from bovine liver, for example, are in the range of 10–20 µmol h^-1^ mg^-1^ at 37°C (e.g., Coleman 1970; Anderson and Desnick 1979; Kreutzer et al. 1977). A value of ∼17 µmol h^-1^ mg^-1^ (at 37°C) has been reported also for the only other studied insect PBGS, the bacterially expressed *Drosophila* enzyme, when examined at its (unusual) pH optimum of ∼8 (Kundrat et al. 2003). On the other hand, the hexameric PBGS from *Rhodobacter capsulatus* that also shows a basic pH optimum (Bollivar et al. 2004) displays a specific activity of 450 µmol h^-1^ mg^-1^ that is much higher than that of the *Pieris* enzyme reported here. If confirmed, the comparably high specific activity of *Pieris* PBGS might have evolved to compensate for the low turnover number typical for PBGS enzymes (Jaffe 2004) in a species that produces significant amounts of tetrapyrroles during relative short developmental periods (Kayser 1984; Kayser and Krull-Savage 1984).

Levulinic acid and succinylacetone are well characterized substrate-analogous inhibitors of PBGS from various organisms (Nandi and Shemin 1967; Ebert et al. 1979; Brumm and Friedman 1981; Tschudy et al. 1981; Sassa and Kappas 1983). Both inhibitors are like ALA in that they bind to a conserved lysine at one of the two active sites, known as the P-site, as visualized by crystal structure analysis (Erskine et al. 2001). As proposed by others (Stauffer et al. 2001), levulinic acid competes with ALA for binding to the second site, the A-site, resulting in the competitive behaviour observed with the *Pieris* enzyme. These authors further propose that in the case of uncompetitive or mixed inhibition, as was the case with succinylacetone in the present study, the inhibitor also competes at the P-site or at both sites. Succinylacetone was by about three orders of magnitude more active than levulinic acid with the *Pieris* PBGS in accordance with other studies (Tschudy et al. 1981). This may be due to firmer binding of succinylacetone obviously due to conformational changes, as X-ray studies revealed (Erskine et al. 2001). This effect is unique to succinylacetone and may be the cause for the irreversible inhibition of the enzyme, as described by Tschudy et al. (1981). On the other hand, succinylacetone, as a structural analog of ALA, has also been described as a competitive inhibitor of PBGS (Brumm and Friedman 1981; Sassa and Kappas 1983). A sound interpretation of the present results of inhibition by succinylacetone may be further limited by the possibility of the non-enzymatically formation, under assay conditions, of a mixed pyrrole between succinylacetone and ALA that is even more inhibitory than succinylacetone itself (Brumm and Friedmann 1981). A competitive inhibitor of yet unknown identity has recently been found to accumulate in pupae of *P*. *brassicae* shortly before adult emergence (Kayser et al. 2005).

### Comparative immunological properties of *Pieris* PBGS

While the structures of the PBGS monomers and their homo-octameric enzymatically active complexes are highly conserved, their identities at the sequence level may be as low as ∼35% in distantly related species (Jaffe 2004). The amino acids relevant for catalytic activity are located within the C-terminal α, β-barrel domain of the protein and relatively phylogenetically invariant. Residues at the surface, like those of the N-terminal sequence that is involved in dimer contacts, are more varied. The surface-exposed residues may therefore offer a variety of potential immunological epitopes that display phylogenetic differences among PBGS enzymes.

Not surprisingly, the antiserum against PBGS from *Pieris* did not recognize PBGS from the vertebrates. However, less expectedly, it did not significantly recognize the protein from the non-lepidopteran insects tested, including representatives from Diptera, Hemiptera and Hymenoptera. Among the lepidopteran families, PBGS from three nymphalid butterflies was fully recognized by the antiserum against the *Pieris* enzyme. On the other hand, PBGS from three noctuid moths did not cross react to a significant extent thus displaying a relatively high phylogenetic distance between moths and butterflies (cf., Scoble 1992). More surprising was the heterogeneous behaviour of PBGS from the examined pyralid and tortricid species covering a wide range of immunological response from fairly high to complete lack of cross reaction, possibly reflecting an evolutionary very basic position of these families relative to the other Lepidoptera.

From the evolutionary perspective that flies and butterflies are only distantly related, it is not surprising that, as discussed above, the *Drosophila* PBGS displays some remarkable differences to the *Pieris* counterpart and, furthermore, that PBGS from the housefly was not recognized by the antiserum against the butterfly enzyme. In fact, the most conserved features of PBGS that are in the active site, are situated in the interior of the oligomeric enzyme complex.

## Abbreviations

PBGS -: porphobilinogen synthase
ALA -: 5-aminolevulinic acid
